# A novel method for modelling interaction between categorical variables

**DOI:** 10.1007/s00038-016-0902-0

**Published:** 2016-10-28

**Authors:** Manfred te Grotenhuis, Ben Pelzer, Rob Eisinga, Rense Nieuwenhuis, Alexander Schmidt-Catran, Ruben Konig

**Affiliations:** 1 Radboud University, Nijmegen, The Netherlands; 2 Stockholm University, Stockholm, Sweden; 3 University of Cologne, Cologne, Germany

## Introduction

Sweeney and Ulveling (1972) introduced weighted effect coding, where the estimates for categories of nominal and ordinal variables are deviations from the arithmetic mean, typically from a sample. This somewhat neglected parameterization is preferred over the well-known effect coding (ANOVA) if the data are unbalanced (i.e., when categories hold different numbers of observations) and was recently revived in this journal (te Grotenhuis et al. 2016). In this paper, we show that weighted effect coding can also be applied to regression models with interaction effects. The weighted effect coded interactions represent the additional effects over and above the main effects obtained from the model without these interactions. This is a useful alternative to effect coding when the data are unbalanced as in most observational data. In this contribution, we describe this novel parameterization and provide syntax, data, and examples in SPSS, R, and Stata on http://www.ru.nl/sociology/mt/wec/downloads. For didactical reasons we apply OLS regression models, but weighted effect coded interactions can be used in any generalized linear model. Throughout this text we use the word ‘interaction’, while other researchers prefer ‘moderation’.

### Interactions between categorical variables

#### Dummy coded interaction

When directional interaction hypotheses are tested and categorical (i.e., ordinal or nominal scaled) predictor variables are involved, dummy coding is often appropriate. In this parameterization the main effects relate to a particular subset of respondents and for the remaining subsets the dummy coded interaction effects reflect deviations from these main effects. To create dummy coded interaction variables one has to multiply the original, 0/1 coded, dummy variables (Hardy 1993). As an empirical example we will investigate to what extent the mean BMI differs across three age categories in a group of respondents with one or more children and in a childless group (Umberson et al. 2011). We use data on self-reported body length and weight, in three random samples (*n* = 3314) drawn from the Dutch population (aged 18–70) in 2000, 2005, and 2011 (Eisinga et al. 2002, 2012a, b). We created the dummy coded variables Childless_dc_ with code 1 for respondents with no children and code 0 for respondents with one or more children, Middle_dc_ (code 1 for the middle-aged and 0 for both young and older respondents) and Older_dc_ (1 for older and 0 for both young and middle-aged respondents). The dummy coded interaction variables Childless_dc_ × Middle_dc_, and Childless_dc_ × Older_dc_ are multiplications of these dummy coded variables (see Table 1 and our website for details). First, we estimated the main effects without interaction (see Table 4, Model 1) and second, we added the two interaction variables (Table 4, Model 2). Note that the reference categories (a) respondents with children, (b) youngsters, and (c) childless youngsters are omitted from the two models, which means that their estimates are set to zero.

**Table 1:** Coding scheme for the dummy coded main and interaction effects for the childless, middle-aged and older-aged (references/omitted categories are with children, young, and childless × young)

Categories	Dummy coding				
	Main effects			Interactions	
	Childless_dc_	Middle_dc_	Older_dc_	Childless_dc_ × Middle_dc_	Childless_dc_ × Older_dc_
With children and younger	0	0	0	0	0
With children and middle-aged	0	1	0	0	0
With children and older	0	0	1	0	0
Childless and younger	1	0	0	0	0
Childless and middle-aged	1	1	0	1	0
Childless and older	1	0	1	0	1

Our results show that without interaction, the estimated mean BMI among childless respondents is a significant 0.9 BMI points lower compared to respondents with children, taking into account their age. Further, the estimated mean BMI is significantly higher in both the middle-aged group (1.36) and in older respondents (2.09), compared to youngsters while controlling for having children or not.

After adding the interactions, the main effect of the dummy coded variable Childless relates to the youngest group only. So, respondents who are youngest and childless have an estimated mean BMI of −1.92 points lower compared to the youngest respondents with children. Further, the main effects in the middle-aged and older group pertain to the respondents with children only. The middle-aged people with children have an estimated mean BMI that is 0.46 (non-significant) higher compared to youngsters with children. The older respondents with children have a BMI that is about 1 BMI point higher (1.22), again compared to youngsters with children.

The two interaction effects (one of them being significant) show the extra effect on BMI on top of the aforementioned main effects. For instance, childless middle-aged respondents have an estimated mean BMI that is −1.92 + 1.22 = −0.7 BMI points less compared to middle-aged respondents with children. Likewise, the childless middle-aged respondents have an estimated mean BMI that is 1.68 higher (0.46 + 1.22) compared to youngsters who are childless.

#### Effect coded interaction

It seems a bit odd to use dummy coding in our example because to our knowledge there is no theory that for instance predicts a stronger age-effect among the childless or a weaker effect of having children among the middle-aged. In general, dummy coding is less appropriate if one is agnostic about the direction of effects as the selection of reference categories and the associated statistical tests are then mostly arbitrary. One popular solution is effect coding, where in interaction models the main effect represents a grand mean effect while the interaction effects are deviations from that grand mean effect. This grand mean effect is unweighted, so effect coding is tailor-made for so-called completely balanced designs (Berger and Wong 2009). In such designs all cells have equal numbers of observations. This is not a necessary condition for the sample data; it suffices to assume a population with such a balanced design, while the sample is unbalanced due to randomness for instance. Especially in experimental settings where equal group sizes are often desired, this type of parameterization is well suited to test whether the treatment effect differs across relevant groups (Berger and Wong 2009). Note that in that particular case there are no hypotheses about the directions of the interaction effects.

In general, an effect coded variable has code 1 for a specific category, 0 for all other categories save the statistically redundant and, therefore, omitted reference category, which is coded −1 (Hardy 1993). In our example, we created six effect coded interactions which are the result of the effect coded variables Childless_ec_ and With Children_ec_ multiplied with Young_ec_, Middle_ec_, and Older_ec_, which are also effect coded (see Table 2 and our website for details).

**Table 2:** Coding scheme for the effect coded main and interactions effects for the childless, middle-aged and older-aged (omitted categories are with children, young, and childless × young)

Categories	Effect coding				
	Main effects			Interactions	
	Childless_ec_	Middle_ec_	Older_ec_	Childless_ec_ × Middle_ec_	Childless_ec_ × Older_ec_
With children and younger	−1	−1	−1	1	1
With children and middle-aged	−1	1	0	−1	0
With children and older	−1	0	1	0	−1
Childless and younger	1	−1	−1	−1	−1
Childless and middle-aged	1	1	0	1	0
Childless and older	1	0	1	0	1

The results for this effect coded interaction model are given in Table 4, and again model 1 with no interaction is presented first. The grand mean BMI is 24.73 (intercept) and respondents with children have an estimated mean BMI of 24.73 **+** 0.45 = 25.18. The respondents with no children have an estimated mean BMI of 24.73 − 0.45 = 24.28. To find the grand mean again we have to sum 25.18 and 24.28 and divide it by 2, resulting in 24.73, which again is the value for the intercept. This proves that with regard to the point of reference, effect coding does not take into account the possible unequal number of observations in the categories. Compared to this grand mean of 24.73, the estimated mean BMI is −1.15 lower for the younger respondents, 0.21 higher for the middle-aged, and 0.94 higher for the older respondents. Note that when these three deviations are summed, the outcome equals zero, which is typical for using a balanced design. After adding the effect coded interaction variables (Model 2), the grand mean shifts to 24.88 and the main effects also change. This is due to the unbalanced nature of our data, for instance the number of older respondents without children is 62, whereas 3314/6 = 552 is expected in a completely balanced design. The interaction effects denote the extra change in the estimated mean BMI over and above the unweighted main effect. Young, childless respondents have a mean BMI of 0.41 less, so their estimated mean BMI is 24.88 − 0.56 − 0.97 − 0.41 = 22.94. For youngsters with children the estimated mean is 0.41 higher than the main effects indicate: 24.88 + 0.56 − 0.97 + 0.41 = 24.88. Note that both interaction effects are counterparts and note also that the sum of all interaction effects equals zero, which again is the result of assuming balanced data.

#### Weighted effect coded interaction

In our example the sample data are far from being balanced (see the numbers of observation per category in Table 4). This means that testing interaction effects under the assumption of balanced data with a grand mean effect as a point of reference is less appropriate, because most probably the data are not balanced in the target population as well. In such cases testing interaction effects against the effects found without interactions makes more sense, as the latter are overall main effects, taking into account the numbers of observation per category. This is a new way of modelling interaction using weighted effect coded interaction variables. Unlike dummy coding and effect coding, these interaction variables are not simply the multiplication of two weighted effect coded variables. Instead, weights are assigned to the interaction variables to obtain main effects that equal the effects from the model without these interactions (see Table 3 for details and our website for in-depth matrix information and for syntax in SPSS, R and Stata). The orthogonal interaction effects then denote the extra effect over and above the main effects found in the model without these interactions, no matter whether the data are unbalanced or not. In case the data are completely balanced, the estimates from weighted effect coding are equal to those from effect coding, but they can be quite different in effect size and associated *t* values when the data are unbalanced. This is illustrated in Table 4, last two columns. In Model 1 (without interactions), the estimate for the intercept equals 24.98, and equals the observed (arithmetic) sample mean in our dataset. Respondents with children have an estimated mean BMI that is 0.29 higher than 24.98, whereas childless respondents score 0.61 BMI points lower. Further, the youngster have a mean BMI of 24.98 − 1.24 = 23.74, whereas for the middle-aged the mean BMI is slightly higher (+0.12), and finally for older respondents we must add 0.85 to 24.98 to find their estimated mean BMI. Note that the effects no longer add up to 0, as we take into account the unequal numbers of observations. When the six interactions are added in Model 2, nothing changes in the intercept or main effects, because the interactions have a mean of 0 and are orthogonal to the main effects. The interpretation of these interactions is straightforward: it is the extra estimated mean BMI over and above the main effects found in the model without interactions. For instance the young respondents with no children have a BMI which is an extra −0.17 lower compared to 24.98 (on top of the main effects −1.24 and −0.61). Note that the equal sized interaction effects −0.17 (childless × young) and 0.17 (childless × older) have quite different *t* values (−2.75 vs. 0.39). This is a direct result of taking into account the different number of respondents per category; there are much less older people than younger people, so the power of that test is lower. Note also that the *t* value for children × older and childless × older is equal (−0.39) as the dichotomy children/childless is mutually exclusive. Note further that weighted effect coded interaction effects do not add up to zero as in effect coding, again due to the different numbers per category. We finally add that in Table 4 the explained variances are the same in all three models 1 and in all three models 2. So, no matter which type of coding is used, the predicted BMI scores are exactly the same. The only difference is the type of base line one wishes to use. In dummy coding this base line is a particular subset of respondents, in effect coding it is a grand mean of estimates (neglecting the possible unbalance in the data), while in weighted effect coding the base line is the weighted main effect.

**Table 3:** Coding scheme for the *weighted effect coded* main and interactions effects for the childless, middle-aged and older-aged (omitted categories are with children, young, and childless × young)

Categories	Weighted effect coding				
	Main effects			Interactions	
	Childless_wec_	Middle_wec_	Older_wec_	Childless_wec_ × Middle_wec_	Childless_wec_ × Older_wec_
With children and younger	−(*n*_c_/*n*_w_)	−(*n*_m_/*n*_y_)	−(*n*_o_/*n*_y_)	(*n*_cm_/*n*_wy_)	(*n*_co_/*n*_wy_)
With children and middle-aged	−(*n*_c_/*n*_w_)	1	0	−(*n*_cm_/*n*_wm_)	0
With children and older	−(*n*_c_/*n*_w_)	0	1	0	−(*n*_co_/*n*_wo_)
Childless and younger	1	−(*n*_m_/*n*_y_)	−(*n*_o_/*n*_y_)	−(*n*_cm_/*n*_cy_)	−(*n*_co_/*n*_cy_)
Childless and middle-aged	1	1	0	1	0
Childless and older	1	0	1	0	1

*n*_*w*_ number of observations (*n*) in category with children*, n*_*c*_ *n* in category childless*, n*_*y*_ *n* in category young*, n*_*m*_ *n* in category middle*, n*_*o*_ *n* in category older*, n*_*wy*_ *n* in category with children and young*, n*_*wm*_ *n* in category with children and middle*, n*_*wo*_ *n* in category with children and older*, n*_*cy*_ *n* in category childless and young*, n*_*cm*_ *n* in category childless and middle*, n*_*co*_ *n* in category childless and older

**Table 4:** Ordinary least squares regression effects on the body mass index (BMI), using dummy coding, effect coding, and weighted effect coding, without interactions (Model 1) and with interactions (Model 2), number of cases per category between brackets (*n*) Data source: Eisinga et al. (2002, 2012a, b), total *n* = 3314

OLS effects on BMI	Dummy coding		Effect coding		Weighted effect coding	
	b-estimates	*t* values	b-estimates	*t* values	b-estimates	*t* values
**Model 1**						
Intercept	24.02	118.58	24.73	317.14	24.98	389.21
Having children						
With children (2254)	0.00 (ref)		0.45	5.51	0.29	5.51
Childless (1060)	−0.90	−5.51	−0.45	−5.51	−0.61	−5.51
Age-group						
Young (610)	0.00 (ref)		−1.15	−8.46	−1.24	−7.81
Middle (2111)	1.36	6.93	0.21	2.28	0.12	2.38
Older (593)	2.09	8.56	0.94	7.52	0.85	5.99
Variance explained	6.1 %		6.1 %		6.1 %	
**Model 2**						
Intercept	24.88	67.06	24.88	223.52	24.98	389.54
Having children						
With children (2254)	0.00 (ref)		0.56	4.99	0.29	5.51
Childless (1060)	−1.92	−4.74	−0.56	−4.99	−0.61	−5.51
Age-group						
Young (610)	0.00 (ref)		−0.97	−5.97	−1.24	−7.80
Middle (2111)	0.46 ns	1.21	0.11 ns	0.87	0.12	2.39
Older (593)	1.22	3.01	0.86	4.72	0.85	5.99
Children × young (99)	Not applicable		0.41	2.50	0.86	2.75
Children × middle (1624)	Not applicable		−0.21 ns	−1.66	−0.05	−2.00
Children × older (531)	Not applicable		−0.20 ns	−1.10	−0.02 ns	−0.39
Childless × young (511)	0.00 (ref)		−0.41	−2.50	−0.17	−2.75
Childless × middle (487)	1.22	2.72	0.21 ns	1.66	0.15	2.00
Childless × older (62)	1.21 ns	1.88	0.20 ns	1.10	0.17 ns	0.39
Variance explained	6.3 %		6.3 %		6.3 %	

*ns* not significant (*t* value <1.96), *t* values are presented for illustrative purposes

To save space we did not include control variables in our models, the interpretation, however, is basically the same: the weighted effect coded interaction effects still reflect deviations from the weighted main effects, only this time after taking into account one or more control variables. Because the weighted effect coded interactions may be correlated with the control variable(s), the main effects in a controlled model with and without weighted coded interaction parameters can be different in such cases (see our website for an example). The interaction between weighted effect coded variables and interval/ratio scaled variables is available on our website as well.

To conclude: whenever non-directional interaction hypotheses are tested using unbalanced data and this unbalancedness is deemed relevant for the target population, weighted effect coded interactions are to be preferred over effect coded interactions.

### Weighted effect coded interactions in generalized linear models

In this contribution, we showed that weighted effect coded interaction effects represent deviations from the overall main effects (i.e., the main effects found in a model without interaction). This general interpretation holds for any generalized linear model. However, we must add that in logistic regression models the main and interaction effects relate to the odds (i.e., *p*_1_/(1 **−** *p*_1_)). They do not directly relate to *p*_1_ itself, i.e., the estimated probability to score 1 on the dependent variable. In fact, even without interaction parameters, the effects of the predictor variables in a logistic regression model exhibit interaction when the probability (*p*_1_) is considered (Mood 2010).
